# Role of Nitric Oxide Synthases in Respiratory Health and Disease: Insights from Triple Nitric Oxide Synthases Knockout Mice

**DOI:** 10.3390/ijms25179317

**Published:** 2024-08-28

**Authors:** Takaaki Ogoshi, Kazuhiro Yatera, Hiroshi Mukae, Masato Tsutsui

**Affiliations:** 1 Department of Respiratory Medicine, Kokura Memorial Hospital, 1-1 Asano, Kokura-kita-ku, Kitakyushu 803-0802, Japan; togoshi@med.uoeh-u.ac.jp; 2 Department of Respiratory Medicine, University of Occupational and Environmental Health, Japan, 1-1 Iseigaoka, Yahata-nishi-ku, Kitakyushu 807-8555, Japan; yatera@med.uoeh-u.ac.jp; 3 Department of Respiratory Disease, Nagasaki University Graduate School of Biomedical Sciences, Nagasaki 852-8501, Japan; hmukae@nagasaki-u.ac.jp; 4 Department of Pharmacology, Graduate School of Medicine, University of the Ryukyus, 207 Uehara, Nishihara, Okinawa 903-0215, Japan

**Keywords:** asthma, bone marrow, mice, nitrate, nitric oxide, nitric oxide synthases, pulmonary emphysema, pulmonary fibrosis, pulmonary hypertension

## Abstract

The system of nitric oxide synthases (NOSs) is comprised of three isoforms: nNOS, iNOS, and eNOS. The roles of NOSs in respiratory diseases in vivo have been studied by using inhibitors of NOSs and NOS-knockout mice. Their exact roles remain uncertain, however, because of the non-specificity of inhibitors of NOSs and compensatory up-regulation of other NOSs in NOS-KO mice. We addressed this point in our triple-n/i/eNOSs-KO mice. Triple-n/i/eNOSs-KO mice spontaneously developed pulmonary emphysema and displayed exacerbation of bleomycin-induced pulmonary fibrosis as compared with wild-type (WT) mice. Triple-n/i/eNOSs-KO mice exhibited worsening of hypoxic pulmonary hypertension (PH), which was reversed by treatment with sodium nitrate, and WT mice that underwent triple-n/i/eNOSs-KO bone marrow transplantation (BMT) also showed aggravation of hypoxic PH compared with those that underwent WT BMT. Conversely, ovalbumin-evoked asthma was milder in triple-n/i/eNOSs-KO than WT mice. These results suggest that the roles of NOSs are different in different pathologic states, even in the same respiratory diseases, indicating the diversity of the roles of NOSs. In this review, we describe these previous studies and discuss the roles of NOSs in respiratory health and disease. We also explain the current state of development of inorganic nitrate as a new drug for respiratory diseases.

## 1. Introduction

The global prevalence of respiratory diseases, including chronic obstructive pulmonary disease (COPD), pulmonary fibrosis, pulmonary hypertension (PH), and asthma, is on the rise, imposing significant socio-economic burdens [1,2]. Advancing our understanding of the underlying pathophysiological mechanisms of these diseases is critical for developing novel preventive and therapeutic strategies.

**Figure 1:**
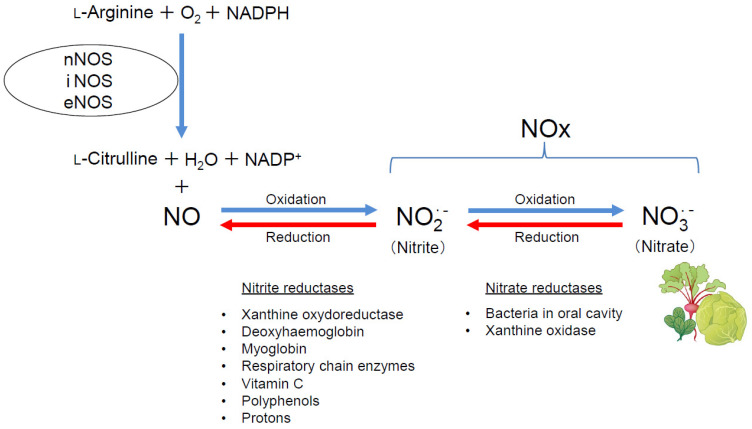
Schematic diagram showing the chemical reactions catalyzed by NOSs. NO is synthesized from L-arginine, oxygen, and NADPH by NOSs along with generation of L-citrulline, water, and NADP^+^. NO is oxidized to nitrite (NO_2_^−^), and, in turn, to nitrate (NO_3_^−^). Nitrate is rich in green leafy vegetables, such as beetroot, lettuce, and spinach. Nitrate is reduced to nitrite by bacteria in the oral cavity and xanthine oxidase, and nitrite is reduced to NO by xanthine oxidoreductase, deoxyhemoglobin, myoglobin, respiratory chain enzymes, vitamin C, Polyphenols, and protons.

Nitric oxide (NO), a gaseous free radical produced from L-arginine by NO synthases (NOSs), is essential for the maintenance of respiratory homeostasis [3,4,5] (Figure 1). The NOSs family is comprised of three isoforms (neuronal NOS [nNOS or NOS1], inducible NOS [iNOS or NOS2], and endothelial NOS [eNOS or NOS3]), all of which are expressed in lung tissues under both physiological and pathological conditions [6,7]. nNOS is expressed in the airway epithelium, vascular endothelium, vascular smooth muscle cells (VSMCs), and nerve cells; iNOS is localized in the airway epithelium, vascular endothelium, VSMCs, and alveolar macrophages; and eNOS is located in the vascular endothelium and VSMCs [7,8,9,10]. NO modulates vascular tone, airway tone, fibrosis, and inflammation, and plays a pivotal role in respiratory pathophysiology [11,12,13,14]. Despite the recognized importance of NO in respiratory health and disease, the exact pathogenetic roles of NO/NOSs in respiratory diseases remain to be fully elucidated.

Previous studies have explored the roles of NO/NOSs in vivo by using non-selective inhibitors of NOSs, including *N*^ω^-nitro-L-arginine (L-NNA), *N*^ω^-nitro-L-arginine methyl ester (L-NAME), *N*^G^-monomethyl-L-arginine (L-NMMA), and asymmetric dimethylarginine (ADMA). However, these inhibitors of NOSs have several non-specific actions, such as antagonizing muscarinic acetylcholine receptors, generating superoxide anions, inhibiting cytochrome c reduction, and blocking vascular-endothelium-independent relaxation [15,16,17,18]. We have reported that mouse vascular lesion formation caused by long-term oral treatment with L-NNA, L-NAME, L-NMMA, or ADMA is not mediated by simple inhibition of eNOS, and that activation of the vascular tissue renin–angiotensin system and enhanced oxidative stress are involved in the long-term vascular effects of those inhibitors of NOSs in an eNOS-independent manner [19,20].

**Figure 2:**
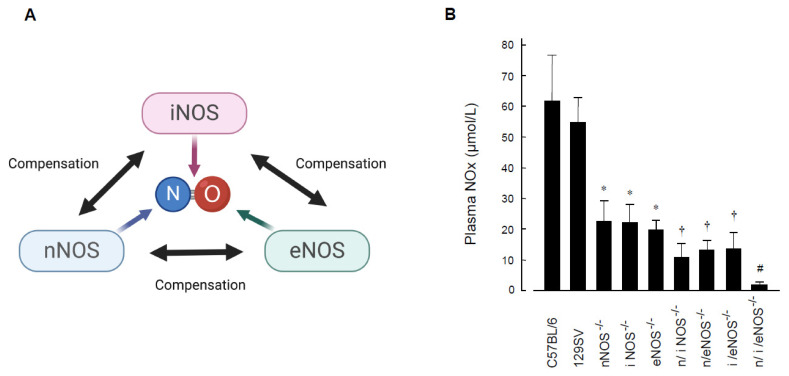
Compensatory interactions among each NOS isoform (**A**) and plasma NOx levels in triple-n/i/eNOSs-KO mice (**B**). (**A**) Schematic diagram showing compensatory interactions among each NOS isoform. (**B**) Plasma NOx levels in wild-type C57BL/6 and 129SV, single-nNOS-KO, -iNOS-KO, -eNOS-KO, double-n/iNOSs-KO, -n/eNOSs-KO, -i/eNOSs-KO, and triple-n/i/eNOSs-KO mice. * *p* < 0.05, ^#^ *p* < 0.01, ^†^ *p* < 0.001 vs. C57BL/6. Data from [28]. Copyright 2005 National Academy of Sciences.

The roles of NO/NOSs in vivo have also been studied by using single-NOS-knockout mice. However, in the single-NOS-knockout model, other NOSs that are not genetically deleted are up-regulated in a compensatory manner (Figure 2A), and phenotypes that originally should be seen are sometimes masked. In fact, it has been shown in eNOS-KO mice that nNOS is up-regulated in the endothelium of the coronary artery [21,22], the cerebral artery [23,24], and the aorta [25,26], and that, even though eNOS-derived NO has a vasodilator effect, endothelium-dependent vasodilation to acetylcholine in the coronary artery and the cerebral artery is not impaired in eNOS-KO mice [22,24]. Furthermore, it has been reported that, even though eNOS-derived NO has an anti-atherosclerotic effect, and even though eNOS-KO mice exhibit accumulation of cardiovascular risk factors resembling human metabolic syndrome [27], eNOS-KO mice do not develop atherosclerotic vascular lesions. Thus, the exact roles of endogenous NO/NOSs in vivo still remain obscure.

One of the most appropriate ways to address this issue is to use a genetically engineered mouse deficient in all three NOS isoforms. We previously succeeded in developing triple-n/i/eNOSs-KO mice by crossing single-NOS-KO mice with each other [28] and have been studying the roles of NOSs in vivo with the use of the triple-mutant mice. There is no protein expression of the three NOSs and no NOSs enzymatic activity in triple-n/i/eNOSs-KO mice, even after lipopolysaccharide administration. Plasma and urinary NOx (nitrite-plus-nitrate) levels, indexes of systemic NO production, are very low (about 3% of the normal value in wild-type [WT] mice) in-triple n/i/eNOSs-KO mice (Figure 2B). While triple-n/i/eNOSs-KO mice are viable and appear to be normal, their survival and fertility rates are low as compared with WT mice. Triple-n/i/eNOSs-KO mice display multiple phenotypes in the cardiovascular system, the renal system, the metabolic system, the central nervous system, the bone system, and the respiratory system, revealing important roles of NOSs in the pathogenesis of a wide variety of disorders [6,29,30,31].

We demonstrated through the use of our triple-n/i/eNOSs-KO mice the preventive role of systemic NOSs in spontaneous pulmonary emphysema [32] and in bleomycin-induced pulmonary fibrosis [33], the protective role of systemic and myelocytic NOSs in chromic hypoxic pulmonary hypertension (PH) [7], and the opposing harmful role of systemic NOSs in ovalbumin-evoked asthma [34]. We also indicated a beneficial effect of oral treatment with sodium nitrate on chronic hypoxic PH in triple-n/i/eNOSs-KO mice. In this review, we describe these previous studies and discuss the roles of NOSs in respiratory health and disease. In addition, we explain the current state of development of inorganic nitrate as a new drug for respiratory diseases.

## 2. Diverse Roles of Nitric Oxide Synthases in Respiratory Diseases

### 2.1. Preventive Role of Nitric Oxide Synthases in Spontaneous Pulmonary Emphysema

Pulmonary emphysema, a key feature of COPD, involves the permanent enlargement of air spaces and alveolar wall destruction [35]. In conjunction with airway thickening, it leads to airflow limitation, impaired gas exchange, and progressive air trapping, particularly during exhalation [36,37]. COPD is caused by chronic exposure to irritating gases or particulate matter, which are most often derived from tobacco smoke. COPD is progressive and worsens by infection, which shortens life span. The prevalence of COPD in people older than 40 years is about 10% [38], and COPD is the third leading cause of death in the world (an estimated 3.3 million deaths). The number of patients with COPD is expected to increase because of population aging and continuation of smoking. Bronchodilators (anti-cholinergic drugs and adrenergic β_2_-receptor stimulants) are used in the treatment of symptomatic patients with COPD. A radical cure has yet to be developed, however, since the exact molecular mechanisms of pulmonary emphysema are not fully understood.

No previous study has reported the role of nNOS in pulmonary emphysema. There are discrepant results in previous studies with iNOS-KO mice. Two studies have reported alleviation of elastase- and tobacco-smoke-induced pulmonary emphysema in iNOS-KO mice compared with WT mice [39,40], whereas another study reported a similar degree of elastase-induced pulmonary emphysema in iNOS-KO and WT mice [41]. As for eNOS, a comparable degree of elastase-induced pulmonary emphysema in eNOS-KO and WT mice has been indicated [41].

Chronic treatment with the inhibitor of NOSs, L-NAME, mitigates cigarette-smoke-induced pulmonary emphysema in mice [42]. However, chronic treatment with the NO precursor L-arginine also similarly reduces cigarette-smoke-induced pulmonary emphysema in mice. These results suggest that the effects of L-NAME on cigarette-smoke-induced pulmonary emphysema are not mediated by inhibition of NOSs. In agreement with this study, it has been shown that chronic treatment with L-NAME does not inhibit eNOS activity [43].

**Figure 3:**
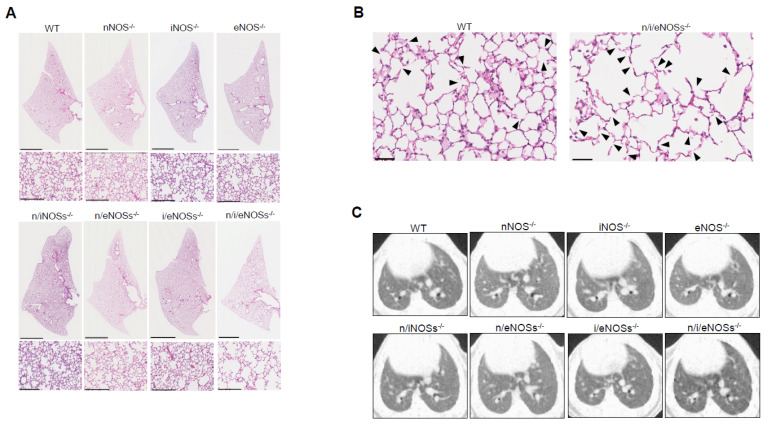
Spontaneous pulmonary emphysema in triple-n/i/eNOSs-KO mice. (**A**) Hematoxylin and eosin staining of lung tissues. Scale bars in upper and lower pictures indicate 2500 and 250 μm, respectively. (**B**) Hematoxylin and eosin staining of lung tissues in WT and triple-n/i/eNOSs-KO mice. Arrowheads indicate destroyed alveoli. Scale bar = 50 μm. (**C**) Microscopic computed tomography images of the lung. Eight-week-old male WT mice, three strains of single-NOS-KO mice, three strains of double-NOSs-KO mice, and triple-n/i/eNOSs-KO mice were studied. Data from [32].

We compared lung morphology in WT mice, three strains of single-NOS-KO mice, three strains of double-NOSs-KO mice, and triple-n/i/eNOSs-KO mice [32]. A histopathological examination and microscopic computed tomography (CT) analysis revealed pulmonary emphysematous changes in triple-n/i/eNOSs-KO mice (i.e., augmented alveolar destructive index, reduced lung field CT value, and larger end-expiratory lung volume) as compared with WT mice (Figure 3A–C). These pulmonary emphysematous changes were detected in triple-n/i/eNOSs^−/−^ mice from 4 weeks after birth. In contrast, no such changes were seen in any single-NOS-KO or double-NOSs-KO mice.

Cap analysis of gene expression (CAGE) was performed in order to examine molecular mechanisms. Gene ontology (GO) term enrichment analysis and Kyoto Encyclopedia of Genes and Genomes (KEGG) pathway analysis indicated that the Wnt/β-catenin pathway is down-regulated in lung tissues of triple-n/i/eNOSs-KO mice as compared with WT mice. mRNA expression levels of 13 genes related to the Wnt/β-catenin pathway were decreased in lung tissues of triple-n/i/eNOS-KO mice.

Down-regulation of the Wnt/β-catenin pathway is thought to play a pathogenetic role in pulmonary emphysema as follows: first, expression levels of the Wnt ligands and Wnt/β-catenin signaling components are reduced in lung tissues of mice with elastase- and tobacco-smoke-induced pulmonary emphysema [44]; second, treatment with lithium chloride, which is an activator of the Wnt/β-catenin pathway, restores expression levels of the Wnt ligands and Wnt/β-catenin signaling components and reverses pulmonary emphysema in those mice [44]; third, decreased Wnt4 levels, augmented β-catenin degradation, and lung morphological abnormality (focal distal airway dilatation) that mimic pulmonary emphysema are observed in human patients bearing a loss-of-function Wnt4 gene mutation [45]. It is thus likely that down-regulation of the Wnt/β-catenin pathway is involved in the occurrence of spontaneous pulmonary emphysema in triple-n/i/eNOSs-KO mice.

It is conceivable, based on these findings, that NOSs play a preventive role in the development of spontaneous pulmonary emphysema in mice in vivo.

### 2.2. Protective Role of Nitric Oxide Synthases in Pulmonary Fibrosis

Pulmonary fibrosis is a serious parenchymal lung disease with a poor prognosis. Pulmonary fibrosis is characterized by progressive scarring and thickening of the lung interstitium (alveolar walls and septa and peri-vascular, peri-lymphatic, and peri-bronchiolar connective tissues) [46]. Although inflammation is thought to initiate pulmonary fibrosis, its exact molecular mechanism has yet to be clarified.

The role of nNOS in pulmonary fibrosis has not been reported in any previous study. eNOS appears to play a protective role in pulmonary fibrosis because eNOS-KO mice display impaired repair of bleomycin-induced pulmonary fibrosis [47] and because eNOS-overexpressing transgenic mice exhibit milder bleomycin-induced pulmonary fibrosis [48]. There are conflicting results about the role of iNOS. It has been reported that iNOS-KO mice and the administration of iNOS inhibitors in mice attenuate bleomycin-induced pulmonary fibrosis and lung injury [49,50], whereas, in contrast, worsening of bleomycin-induced lung injury in iNOS-KO mice has also reported [51].

Treatment with L-NAME significantly worsened the mortality rate in bleomycin-treated WT mice (47.4% [9/19] without L-NAME vs. 87.5% [7/8] with L-NAME). However, since only one bleomycin-treated and L-NAME-treated WT mouse survived, a comparison was not made of the degree of pulmonary fibrosis with and without treatment with L-NAME [50].

**Figure 4:**
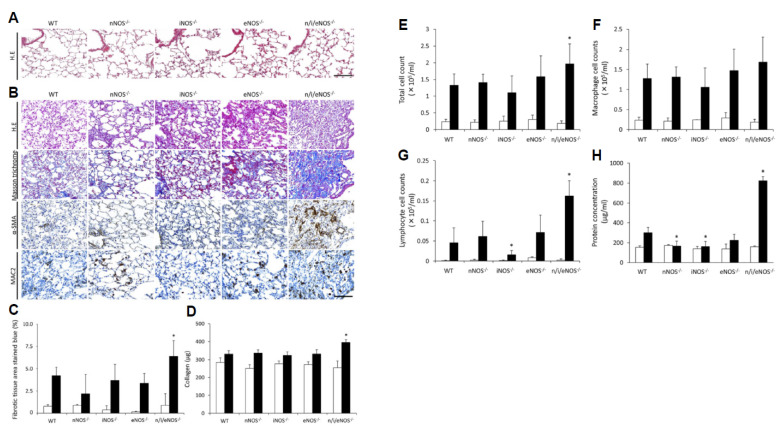
Deterioration of pulmonary fibrosis and increased inflammatory cell number in the bronchoalveolar lavage fluid in triple-n/i/eNOSs-KO mice at 2 weeks after bleomycin treatment. (**A**) Hematoxylin–eosin (H.E) staining in normal saline-treated mice. Scale bar = 100 μm. (**B**) Hematoxylin–eosin staining, Masson’s trichrome staining, α-smooth muscle actin (SMA) staining, MAC-2 staining in bleomycin-treated mice. Scale bar = 100 μm. (**C**) Fibrotic tissue area (blue-stained). (**D**) Collagen content in lung tissues. White and black bars indicate normal saline-treated (n = 3) and bleomycin-treated mice (n = 5), respectively. * *p* < 0.05 vs. bleomycin-treated WT mice. (**E**–**H**) Total, macrophage, and lymphocyte cell counts and total protein concentrations in the bronchoalveolar lavage fluid. White and black bars indicate normal saline-treated (n = 3) and bleomycin-treated mice (n = 5), respectively. * *p* < 0.05 vs. bleomycin-treated WT mice. Data from [33].

We compared the degree of pulmonary fibrosis and inflammatory changes in WT mice, each single-NOS-KO mouse, and triple-n/i/eNOSs-KO mice at 2 weeks after bleomycin treatment [33]. In the above study with L-NAME, 1 U/body/day of bleomycin was administered intraperitoneally every other day four times, while, in our study, 8.0 mg/kg/day of bleomycin was administered intraperitoneally for 10 consecutive days and few mice died throughout the experimental period. At 2 weeks after bleomycin treatment, pulmonary fibrotic tissue area (Figure 4A–C), lung collagen content (Figure 4D), total and lymphocyte cell numbers, and total protein concentrations in the bronchoalveolar lavage fluid (BALF) (Figure 4E–H) were all largest in the triple-n/i/eNOSs-KO mice. These changes were associated with significantly higher levels in lung tissues of proinflammatory cytokines (interleukin-6 [IL-6], IL-1β, tumor necrosis factor-α [TNF-α], C-C chemokine ligand 2 [CCL2], which is also known as monocyte chemotactic protein-1 [MCP-1], and transforming growth factor-β1 [TGF-β1]). TGF-β1 produces connective tissue growth factor (CTGF) [52] and collagen Ⅰ [53], both of which act as downstream mediators of TGF-β1 [54]. Significantly higher CTGF and collagen Ⅰ levels in lung tissues were also noted in triple-n/i/eNOSs-KO mice after bleomycin treatment. It is thus likely that lung inflammation and the TGF-β pathway are involved in the exacerbation of bleomycin-induced pulmonary fibrosis in triple-n/i/eNOSs-KO mice. iNOS-KO mice showed comparable pulmonary fibrotic tissue area (Figure 4A–C) and lung collagen content (Figure 4D) and significantly lower lymphocyte cell number and total protein concentrations in BALF (Figure 4E–H) after bleomycin treatment as compared with WT mice.

Simultaneous oral treatment with the NO donor isosorbide dinitrate restored reduced serum NOx levels and inhibited bleomycin-induced pulmonary fibrosis in triple-n/i/eNOSs-KO mice, suggesting that exacerbation of bleomycin-induced pulmonary fibrosis in triple-n/i/eNOSs-KO mice is indeed caused by defective NO production.

From these findings, it is conceivable that NOSs play a protective role in the pathogenesis of bleomycin-induced pulmonary fibrosis in mice in vivo.

### 2.3. Protective Role of Nitric Oxide Synthases in Pulmonary Hypertension

Pulmonary hypertension (PH) is a disorder of the pulmonary artery microvasculature characterized by elevated pulmonary arterial pressure, leading to right-sided heart failure and early death. In spite of the recent progress in the therapeutic disciplines, the prognosis of patients with PH remains poor, and the survival rate of patients with PH is comparable to or worse than that with all cancers [55]. It is known that bone morphogenic protein receptor 2 (BMPR2) gene mutation, defective NO and prostaglandin I_2_ production, and up-regulation of endothelin-1 and its receptor are linked to the occurrence of PH. Radical therapeutic strategies have yet to be developed, however, since the precise molecular mechanism of PH remains uncertain.

No previous study has reported the role of nNOS in PH. Treatment with the iNOS inhibitor has been shown to reduce chronic hypoxic PH in rats, suggesting the detrimental role of iNOS in chronic hypoxic PH [56]. The role of eNOS in PH is controversial as aggravation of, and, in contrast, alleviation of, chronic hypoxic PH in eNOS-KO mice has been reported [57,58].

Treatment with non-selective inhibitors of NOSs has been reported to increase pulmonary artery pressure in animals with chronic hypoxia-induced PH, whereas there are also previous studies reporting a lack of such increases [12]. Treatment with L-NAME increased pulmonary artery pressure in perfused lungs of PH rats exposed to chronic hypoxia; however, this effect was not reversed by simultaneous treatment with L-arginine, indicating a non-specific effect of L-NAME [12]. 

We performed a clinical study on 36 patients with idiopathic pulmonary fibrosis and a basic study on WT mice, each single-NOS-KO mouse, and triple-n/i/eNOSs-KO mice [6]. In the patients with idiopathic pulmonary fibrosis, NOx levels in BALF were inversely correlated with pulmonary artery systolic pressure measured by Doppler echocardiography, suggesting the presence of decreased NO levels in the lungs of patients with World Health Organization (WHO) classification group 3 PH. In mice exposed to chronic hypoxia, the survival rate and PH (as assessed by elevation of right ventricular systolic pressure, increased right ventricular hypertrophy, and pulmonary artery vascular remodeling) worsened in triple-n/i/eNOSs-KO mice and, to a smaller degree, in eNOS-KO mice as compared with WT mice.

While NO is oxidized to nitrite (NO_2_^−^), and, subsequently, to nitrate (NO_3_^−^), nitrate, conversely, is reduced to nitrite and then to NO through the reverse pathway. In other words, nitrate acts as an endogenous NO donor in the nitrate–nitrite–NO pathway in an NOSs-independent manner [59,60]. It has been reported that nitrate is reduced to nitrite by bacteria in the oral cavity and xanthine oxidase, and nitrite is reduced to NO by xanthine oxidoreductase, deoxyhemoglobin, myoglobin, respiratory chain enzymes, vitamin C, Polyphenols, and protons [61] (Figure 1). Simultaneous treatment with 5 mmol/L sodium nitrate significantly improved decreased plasma NOx levels, reduced survival, and PH in triple-n/i/eNOSs-KO mice after chronic hypoxic exposure. Although simultaneous treatment with 2 and 45 mmol/L sodium nitrate also restored decreased plasma NOx levels, they did not affect survival or PH in triple-n/i/eNOSs-KO mice after chronic hypoxic exposure. These results indicate that there is no dose dependency in the effects of sodium nitrate.

**Figure 5:**
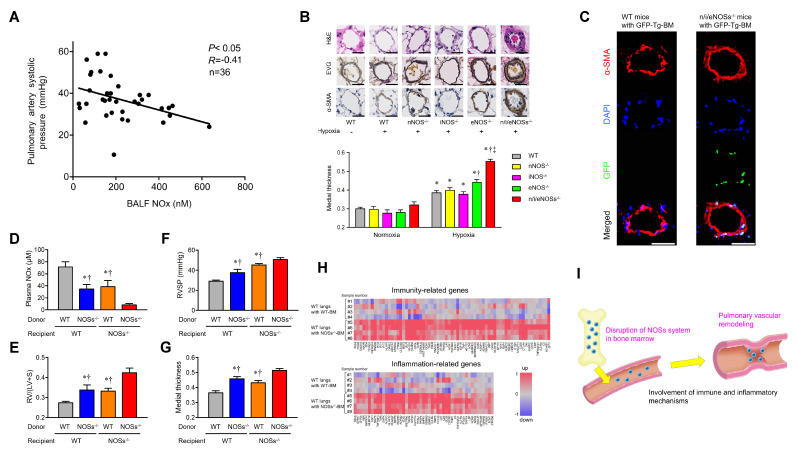
The protective role of NO derived from myelocytic NOSs in chronic hypoxia-induced pulmonary hypertension in mice. (**A**) An inverse correlation between pulmonary artery systolic pressure and NOx (nitrite plus nitrate) levels in bronchoalveolar lavage fluid in patients with idiopathic pulmonary fibrosis. Pulmonary artery systolic pressure estimated by Doppler echocardiography was significantly and negatively correlated with NOx concentrations in bronchoalveolar lavage fluid in patients with idiopathic pulmonary fibrosis. Statistical analysis was performed using the Pearson correlation coefficient. BALF, bronchoalveolar lavage fluid. (**B**) Hematoxylin and eosin staining, elastic van Gieson staining, and α-smooth muscle actin staining of small pulmonary arteries. Scale bar = 50 μm. Medial thickness of small pulmonary arteries (50–150 µm in diameter) (n = 5–10). Statistical analyses were performed by an ANOVA followed by Bonferroni’s post hoc test for multiple comparisons. * *p* < 0.05 vs. normoxia; ^†^ *p* < 0.05 vs. wild type; ^‡^ vs. eNOS^−/−^. H&E, hematoxylin and eosin staining; EVG, elastic van Gieson staining; α-SMA, α-smooth muscle actin staining. (**C**) Immunofluorescent staining in the lungs of wild-type and triple-n/i/eNOSs^−/−^ mice transplanted with bone marrow cells isolated from green-fluorescent-protein-transgenic mice after chronic hypoxic exposure. Green-fluorescent-protein-positive green fluorescence and green fluorescent protein/α-smooth-muscle-cell-double-positive white fluorescence were observed (n = 5). Scale bars = 50 μm. DAPI, 4′,6-diamidino-2-phenylindole (nuclear staining); GFP, green fluorescent protein; α-SMA, α-smooth muscle actin. (**D**–**G**) Plasma NOx levels (**D**), weight ratio of the right ventricle to the left ventricle plus the interventricular septum (RV/[LV + S]) (**E**), right ventricular systolic pressure (RVSP) (**F**), and medial thickness of small pulmonary arteries (50–150 µm in diameter) (**G**) after chronic hypoxic exposure (n = 5–6). Statistical analyses were performed by an ANOVA followed by Bonferroni’s post hoc test for multiple comparisons. * *p* < 0.05 vs. wild-type mice transplanted with wild-type bone marrow cells; ^†^ *p* < 0.05 vs. triple-n/i/eNOSs^−/−^ mice transplanted with triple-n/i/eNOSs^−/−^ bone marrow cells. (**H**) Heat maps of differentially expressed mRNAs categorized as immunity and inflammation in the Subio platform. RNA sequencing was performed to obtain the data. (**I**) A schematic diagram showing the protective role of NO derived from myelocytic NOSs in chronic hypoxia-induced pulmonary hypertension. Data from [6]. Reprinted with permission of the American Thoracic Society. Copyright© 2024 American Thoracic Society. All rights reserved. *The American Journal of Respiratory and Critical Care Medicine* is an official journal of the American Thoracic Society.

Abnormality of the bone marrow (BM) is suggested to be involved in the pathogenesis of PH [62]. However, the role of myelocytic NOSs in PH is unknown. After chronic hypoxic exposure, triple-n/i/eNOSs-KO mice showed an increased number of BM-derived vascular smooth muscle progenitor cells in the blood as compared with WT mice. Green fluorescent protein (GFP)-positive green fluorescence and GFP/α-smooth-muscle-actin-double-positive white fluorescence were detected in the pulmonary artery vascular lesions in triple-n/i/eNOSs-KO mice that underwent GFP-overexpressing BM transplantation (BMT) but not in WT mice that underwent it, suggesting the contribution of BM cells to pulmonary vascular remodeling in triple-n/i/eNOSs-KO mice (Figure 5C). Importantly, in WT mice that underwent triple-n/i/eNOSs-KO BMT compared with those that underwent WT BMT, plasma NOx levels reduced and PH deteriorated, and, in triple-n/i/eNOSs-KO mice that underwent triple-n/i/eNOSs-KO BMT compared with those that underwent WT BMT, plasma NOx levels were elevated and PH was mitigated. These findings provide the first evidence for a protective role of the myelocytic NOSs in hypoxic PH in mice in vivo.

RNA sequencing revealed significant up-regulation of 69 mRNAs associated with immunity and 49 mRNAs associated with inflammation in lung tissues of WT mice that underwent triple-n/i/eNOSs-KO BMT compared with those that underwent WT BMT (Figure 5H), suggesting mediation of immune and inflammatory mechanisms in the aggravation of hypoxic PH caused by triple-n/i/eNOSs-KO BMT.

From these findings, it is conceivable that the systemic and myelocytic NOSs play a protective role in the pathogenesis of chronic hypoxic PH in mice in vivo (Figure 5I).

### 2.4. Opposing Detrimental Role of Nitric Oxide Synthases in Asthma

Asthma represents airflow limitation, airway hyperresponsiveness, bronchial inflammation, excessive mucus secretion, and airway remodeling. Fractional exhaled NO is employed as a biomarker of eosinophilic bronchial inflammation in a clinical setting [63], but the real role of NOSs in asthma is still unknown.

Ovalbumin (OVA)-evoked airway hyperresponsiveness is reduced in nNOS-KO mice compared with WT mice, suggesting a detrimental role of nNOS in asthma [64]. The role of iNOS in asthma is controversial as it has been reported that the degree of OVA-evoked airway inflammation is less in iNOS-KO mice than in WT mice [65], whereas a comparable degree of OVA-evoked airway hyperresponsiveness in iNOS-KO and WT mice has also been indicated [64]. The role of eNOS in asthma is also controversial as it has been reported that OVA-evoked and airway hyperresponsiveness and inflammation are attenuated in eNOS-overexpressing mice [66], whereas OVA-evoked airway hyperresponsiveness is similar between eNOS-KO and WT mice [64].

There are conflicting results on the role of NOSs in asthma as studies have shown that treatment with L-NAME suppresses OVA-evoked eosinophilic airway inflammation [67], whereas the same treatment with L-NAME conversely exacerbates Aspergillus fumigatus-evoked airway hyperresponsiveness and inflammation [68].

**Figure 6:**
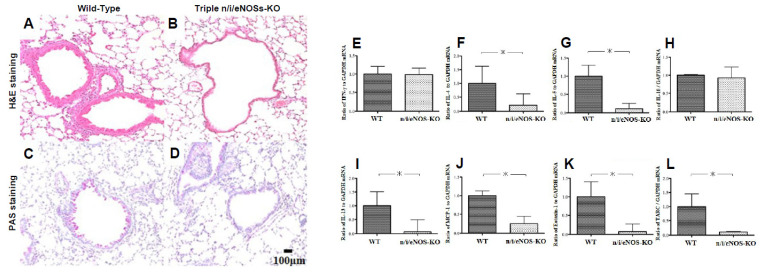
Pathological findings and cytokine and chemokine mRNA expression levels in the lungs of WT and triple-n/i/eNOSs-KO mice that were sensitized and challenged with ovalbumin (OVA). (**A**–**D**) Hematoxylin and eosin staining of lung sections from wild-type (WT) (**A**) and triple-n/i/eNOSs-KO mice (**B**) and periodic acid–Schiff staining of lung sections from WT (**C**) and triple-n/i/eNOSs-KO mice (**D**). (**E**–**L**) mRNA expressions of interferon (IFN)-γ (**E**), interleukin (IL)-4 (**F**), IL-5 (**G**), IL-10 (**H**), IL-13 (**I**), monocyte chemoattractant protein (MCP)-1 (**J**), eotaxin-1 (**K**), and thymus- and activation-regulated chemokine (TARC) (**L**) in the lungs of WT (n = 4) and triple-n/i/eNOSs-KO mice (n = 4). Data are represented as the ratio to glyceraldehyde 3-phosphate dehydrogenase (GAPDH). * *p* < 0.05 by *t* test. Data from [34].

We examined pathological findings and cytokine expression levels in the lungs of WT and triple-n/i/eNOSs-KO mice that were sensitized and challenged with OVA [34]. OVA-evoked eosinophilic lung inflammation, bronchial thickening, and mucus secretion were reduced in triple-n/i/eNOSs-KO mice compared with WT mice (Figure 6A–D). OVA-evoked mRNA expression levels of IL-4, IL-5, IL-13, MCP-1, eotaxin-1, and thymus- and activation-regulated chemokine (TARC), which are type 2 cytokines and chemokines, were also decreased in triple-n/i/eNOSs-KO mice (Figure 6E–G,I–L). In contrast, OVA-evoked mRNA expression levels of interferon (IFN)-γ and IL-10, which are type 1 cytokines, were comparable in the two mice (Figure 6E,H).

From these findings, NOSs appear to play an opposing detrimental role in the pathogenesis of OVA-evoked asthma in mice in vivo, in contrast to the protective roles of NOSs in spontaneous pulmonary emphysema, pulmonary fibrosis, and PH.

## 3. Current State of Development of Inorganic Nitrate as a New Drug for Respiratory Diseases

Inorganic nitrite and nitrate were considered to be mere inert metabolites of NO in the past. Recent research, however, has discovered that nitrate is converted to nitrite and, in turn, to NO, functioning as an endogenous NO donor in the human body [59,60] (Figure 1). The production of NO from nitrate can be easily reversible because the redox potential of the nitrate/nitrite couple (+420 mV) is relatively close to that of the nitrite/NO one (+380 mV). We revealed that long-term oral treatment with sodium nitrate restores reduced NO production and alleviates survival and hypoxic PH in triple-n/i/eNOSs-KO mice, as mentioned above [7]. Many previous basic studies with respiratory disease models also reported beneficial actions of inorganic nitrate. Based on that background, we retrieved the previous literature and clinical trials by using PubMed and ClinicalTrials.gov, respectively. In this section, we describe and discuss the current state of the development of inorganic nitrate as a new therapeutic agent for respiratory diseases.

Although the potential beneficial actions of inorganic nitrate in patients with COPD have attracted attention, clinical trials have indicated discrepant results and previous meta-analyses have not reached a consensus, while some randomized placebo-controlled clinical trials have recently been performed and an updated meta-analysis of these recent trials has been carried out. Eleven clinical studies (287 patients with COPD) were included in the meta-analysis. Potassium-nitrate-rich beetroot juice, as dietary nitrate supplementation, was used in 10 studies, and sodium nitrate dissolved in a 140 mL NaCl solution was employed in the remaining 1 study. Dietary nitrate supplementation increased plasma NOx concentrations and fractional exhaled NO, ameliorated exercise capacity and vascular endothelial function, and relieved dyspnea in patients with COPD. There were no adverse events after dietary nitrate supplementation. It was concluded that dietary nitrate supplementation could be used as a potential treatment for patients with COPD, especially to increase their exercise capacity [69]. 

Inorganic nitrate is thought to improve exercise capacity by improving vascular endothelial function. Vascular endothelial dysfunction is caused by oxidative stress and inflammation. NO elicits vasodilation, inhibits oxidative stress and inflammation, and prevents vascular endothelial dysfunction. It is likely that dietary nitrate supplementation may ameliorate exercise capacity in patients with COPD by converting to NO in vivo and, in turn, by reducing oxidative stress and inflammation and improving vascular endothelial function [69].

Two different clinical trials examining the effects of nitrate-rich beetroot juice on exercise performance in patients with pulmonary fibrosis have been projected, but subjects have not yet been recruited (ClinicalTrials.gov identification number: NCT06488638). 

An exploratory randomized, double-blind, placebo-controlled crossover study reported the effects of nitrate-rich beetroot juice in patients with WHO classification group 1 pulmonary arterial hypertension (PAH). Oral ingestion of beetroot juice for 1 week significantly increased fractional exhaled NO, alveolar NO concentrations, bronchial NO flux, and plasma and salivary NOx levels in patients with PAH as compared with placebo ingestion. Of importance, beetroot juice ingestion tended to improve right ventricular systolic function in patients with PAH and significantly improved the peak-power-output-to-peak-oxygen-consumption ratio (W peak/VO_2_ peak) in PAH patients, reaching an increase in plasma nitrite > 30% (responders). These results suggest that oral supplementation with nitrate-rich beetroot juice increases pulmonary NO production through the nitrate–nitrite, which may be beneficial in patients with PAH [70]. A clinical trial examining the effect of oral sodium nitrate on the oral and gut microbiome and hemodynamics in adults with PH was planned but was withdrawn because of the COVID-19 pandemic restrictions (ClinicalTrials.gov identification number: NCT02000856). 

There is a lack of evidence supporting the efficacy of nitrate for alleviating lung function or lung morphological abnormality. 

## 4. Concluding Remarks and Future Perspectives

**Figure 7:**
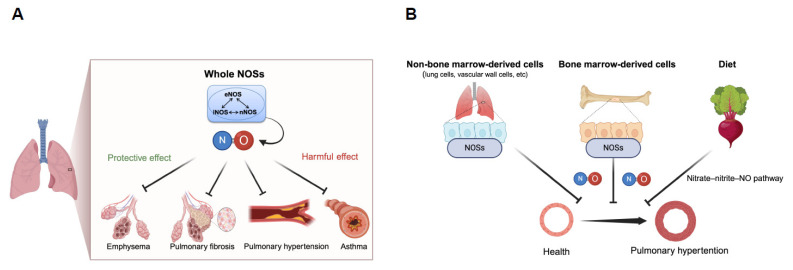
Diverse roles of NOSs in respiratory diseases (**A**) and impact of NOSs in different cell types and dietary nitrate in pulmonary health (**B**). (**A**) Schematic diagram showing the diverse roles of NOSs in respiratory diseases. NOSs play a protective role in the pathogenesis of spontaneous pulmonary emphysema, pulmonary fibrosis, and pulmonary hypertension, whereas they play a harmful role in the pathogenesis of asthma. (**B**) Schematic diagram indicating the impact of NOSs in different cell types and dietary nitrate on pulmonary health. NO derived from NOSs in non-bone-marrow-derived cells, such as lung cells and vascular wall cells, elicits vasodilation and reduces pulmonary vascular resistance, preventing pulmonary hypertension; NO derived from NOSs in bone marrow cells inhibits pulmonary vascular remodeling, preventing pulmonary hypertension; and NO converted from dietary nitrate (e.g., a beetroot, lettuce, and spinach) via the nitrate–nitrite–NO pathway provides an alternative source of NO, maintaining pulmonary health and protecting against the development of pulmonary hypertension.

We demonstrated in studies with triple-n/i/eNOSs-KO mice and with respiratory disease models that systemic disruption of NOSs gives rise to spontaneous pulmonary emphysema possibly through down-regulation of the Wnt/β-catenin pathway; that systemic disruption of NOSs results in exacerbation of bleomycin-induced pulmonary fibrosis in which lung inflammation and the TGF-β pathway are involved; that systemic and myelocytic disruption of NOSs aggravate chronic hypoxic PH that can be reversed by treatment with sodium nitrate; and that systemic disruption of NOSs ameliorates OVA-evoked asthma and lung inflammation. Our findings show that NOSs play protective roles in spontaneous pulmonary emphysema, pulmonary fibrosis, and PH, whereas, in contrast, NOSs play a detrimental role in asthma. It is thus conceivable that the roles of NOSs are different in different pathologic states, indicating the diversity of the roles of NOSs (Figure 7). Our findings should contribute to a better understanding of the roles of NOSs in respiratory health and diseases. Promising results on the beneficial actions of inorganic nitrate supplementation (potassium-nitrate-rich beetroot juice and sodium nitrate) on exercise capacity in patients with COPD and on right ventricular function in patients with PAH have recently been shown. Development of inorganic nitrate as a new therapeutic agent for COPD and PAH is anticipated.
